# Two-Year Healing Success Rates after Endodontic Treatment Using 3D Cleaning Technique: A Prospective Multicenter Clinical Study

**DOI:** 10.3390/jcm11206213

**Published:** 2022-10-21

**Authors:** Giuseppe Pantaleo, Alessandra Amato, Alfredo Iandolo, Dina Abdellatif, Federica Di Spirito, Mario Caggiano, Massimo Pisano, Andrea Blasi, Roberto Fornara, Massimo Amato

**Affiliations:** 1 Department of Medicine and Surgery, University of Salerno, 84081 Salerno, Italy; 2 Department of Endodontics, Faculty of Dentistry, University of Alexandria, Alexandria 21531, Egypt; 3 Department of Periodontics, Faculty of Dentistry, University of Naples, Federico II, 80138 Napoli, Italy; 4 Independent Researcher, 20010 Milan, Italy

**Keywords:** 3D cleaning, NaOCl, ultrasonic, internal heating, healing rate, root canal treatment

## Abstract

Background: Various irrigation techniques for cleansing the endodontic space have been proposed, and internal heating combined with ultrasonic activation (3D cleaning technique) is considered an effective technique. This prospective multicenter clinical study aims to evaluate healing rates for teeth after root canal treatment utilizing the 3D cleaning technique and to report predictive values for success. Material and Methods: Ninety patients referred for a root canal treatment were included. All enrolled patients were treated with the 3D cleaning protocol. Four endodontists performed the clinical procedures and follow-up evaluations. Preoperative, postoperative and follow-up data were gathered from the consented patients. Each patient was assessed for any clinical signs or symptoms. Afterwards, two trained, blinded, and independent evaluators scored the subject’s periapical radiographs. This score was made by checking for the presence or absence of apical periodontitis using the periapical index (PAI). Then, the teeth were classified as healing or healed and were considered a success based on a cumulative success rate of healing. Statistical analysis was performed using the Fisher’s exact test, Pearson correlation, and logistic regression analyses of the preoperative prognostic factors at a 0.05 significance level. Results: 90 patients were evaluated at two years with a follow-up rate of 97.7%. The cumulative success rate of healing was 95.4%. Eight predicting aspects were identified by employing bivariate analyses. Then, using logistic analyses, the two prognostic significant variables directly correlated to healing were the preoperative presence of periapical index (*p* value = 0.016). Conclusions: In this two-year clinical study, the cumulative success rate of healing was 95.4% when patients were treated with the 3D cleaning protocol.

## 1. Introduction

Irrigation in teeth with infected/necrotic pulps aims at dissolving tissue and disrupting biofilms within the primary root canal space and the lateral anatomies of the complex root canal system [1,2,3]. Such lateral anatomies include accessory and lateral canals, loops, isthmuses, ramifications, and microanatomy such as the dentinal tubules. Moreover, remnant tissue and biofilms in such eccentricities act as a nidus for reinfection or persistent infection [4,5]. Generally, use of the conventional syringe-and-needle irrigation is unable to develop adequate shear stress or allow optimal irrigant penetration into dentinal tubules [5,6]. Over the past decade, endodontic research has focused on activated irrigation strategies, wherein techniques such as sonics, ultrasonics, lasers, brushes, and manual dynamic agitation have been investigated with inconclusive results [7,8]. Certainly, some variations could be attributed to variables in the study design, including canal preparation shapes, duration of irrigant agitation, and outcome measures being studied.

However, a less studied approach in historical contexts is the use of warm irrigants such as sodium hypochlorite (NaOCl) to enhance its tissue solvent action [4,6]. Regardless, the rapid buffering of the heat under in vivo conditions may render this technique rather ineffective compared to the results of in vitro studies. An alternative approach is intracanal or internal heating, wherein sodium hypochlorite within root canals is heated using heat carrier tips [9].

Furthermore, an interesting recent study showed an interesting finding that intracanal heating of NaOCl resulted in significantly less debris on the root canal walls compared to irrigation with pre-heated NaOCl [4]. Other studies showed how internal heating combined with ultrasonic activation is considered an effective technique [4,9]. Specifically, this approach (internal heating + ultrasonic activation) is named 3D cleaning.

The current study is the first clinical research that reports the healing rates observed by four independent endodontists utilizing the 3D cleaning protocol.

## 2. Materials and Methods

### 2.1. Study Cohort

The inception cohort comprised 90 patients referred for endodontic treatment in this work. The study protocol for the multicenter, prospective, non-significant risk clinical study was approved by an Institutional Review Board (approval code: 55/21, approval date: 28 April 2021 (University of Naples) and carried out in accordance with the Declaration of Helsinki.

The clinical study evaluated endodontic treatments’ healing rates using the 3D cleaning protocol. Ninety patients met the inclusion criteria. The purpose of the study was explained to the patients, and written informed consent was obtained. All the subjects adhered to previously defined inclusion and exclusion criteria in Table 1. After initiation of the study, the subjects were given the opportunity to withdraw. A total of 90 teeth, one tooth per patient, were treated for the clinical study. All teeth had periapical lesions.

### 2.2. Intervention

Four endodontists participated as investigators in the multicenter, prospective, non-significant risk clinical study to assess the long-term performance of the 3D cleaning protocol. The investigators were trained to use the 3D cleaning protocol and performed a standardized treatment procedure at their independent clinical sites. Using standard coded data sheets, the collected redacted clinical and radiographic data pertained to each treated tooth before (preoperative), and six months and two years after (postoperative) the initial treatment. The data were directly transferred to a database.

### 2.3. Preoperative Data Collection

Prior to treatment, the patients were clinically examined, and radiographs were taken. Pulp and the peri-radicular diagnosis were completed and regarded.

### 2.4. Treatment Procedure

The patients were anesthetized per standard techniques, the injection type being at the endodontist’s discretion. The tooth was isolated with a dental dam. Caries and existing restoration were removed. Missing tooth structures were built up, and conservative straight-line access was performed. Patency was confirmed with #10 and #15 K type hand files (Coltene/Whaldedent Inc., Cuyahoga Falls, OH, USA)) and the working length was achieved using an electronic apex locator (Morita). Teeth were instrumented with a standardized minimal instrumentation protocol that included using hand files up to size ISO #15 and two Hyflex EDM rotary files: 15/03, 20/05 (Coltene/Whaldedent Inc., Cuyahoga Falls, OH, USA) regardless of the initial canal size.

During the shaping phase, 3 mL of 5.25% NaOCl was used between the files using a side-vented 30 G needle (Coltene/Whaldedent Inc., Cuyahoga Falls, OH, USA).

After the shaping phase, a distilled water rinse and then 17% ethylenediaminetetraacetic acid (EDTA, Coltene) were used for 1 min. Then, 5 mL of 5.25% sodium hypochlorite (NaOCl, Coltene/Whaldedent Inc., Cuyahoga Falls, OH, USA) was used, and four cycles of 3D cleaning were performed.

Each cycle consisted of 5 s of internal heating followed by 20 s of ultrasonic activation.

A heating source (System-B, Kerr, CA, USA) was used for the internal heating phase at 180°, 5 mm away from the working length. The tip used was 30/04.

For ultrasonic activation, we used a cordless ultrasonic device with a smooth tip 25/02 (ultrasmart AI, coxo, Fushan, China) 3 mm away from the working length.

Finally, distilled water was used. Canals were subsequently dried with absorbent sterilized paper points. The dried canals were obturated using a modified warm vertical technique with gutta-percha and a biosealer (Bioseal, Coltene/Whaldedent Inc., Cuyahoga Falls, OH, USA). The pulp chamber floor was sealed with bonded composite, and the patients went to the referring dentist for final post-treatment restoration.

### 2.5. Intraoperative Data Collection

Root filling length, sealer extrusion, and coronal seal were documented during the treatment.

### 2.6. Postoperative Data Collection

Post-treatment symptoms were assessed two days after the treatment using a visual analogue scale (VAS; 0 and 10) to rank the level of experienced pain. Each investigator completed a follow-up assessment every three months for patients enrolled at their respective clinical sites. Assessments were standardized and included both clinical and radiographic examinations. The clinical examination involved an update on the medical and dental history, intra-oral evaluation, which included periodontal pocket depth measurements, mobility testing, presence and extent of swelling and soft tissue lesion, and assessment of percussion and palpation.

### 2.7. Outcome Measures and Criteria

Teeth were assessed for healing using a composite endpoint that included clinical and radiographic components. Clinical signs and symptoms, as discussed previously, were utilized for assessing the clinical component. Periapical index scoring (PAI) was utilized to assess the tooth using a periapical radiograph. The scores ranged from 1 (for normal peri-radicular tissue) to 5 (severe periodontitis with exacerbating features).

Based on clinical signs/symptoms and PAI scores, teeth were classified as healed, healing, or diseased [10].

In summary, the diagnosed teeth were classified as follows:

(a) Healed—clinical normalcy accompanied by radiographic PAI scores of 1 or 2.
(b) Healing—clinical normalcy other than tenderness to percussion accompanied by a reduction in the size of peri-radicular lesion or reduction in PAI score.
(c) Failed—the presence of clinical signs and symptoms accompanied by a radiographic PAI score of 3 or higher or an increase in the size of peri-radicular lesion or increase in PAI score.

The teeth classified as healing or healed were considered a success. The combined success of these cases was termed the healing rate.

### 2.8. Calibration of Evaluators

Two experienced endodontists blindly evaluated the radiographs. The images were coded and provided to the evaluators after being randomized between different patients. Before evaluating the images, the two examiners evaluated a series of radiographs independent of the study sample representing a wide range of periapical lesions to account for inter-observer reliability. Cohen’s kappa score was calculated. The exercise was independently performed three times to increase the calibration. In general, each visible root on the radiograph was assigned a PAI score. The highest PAI score for all the roots for a given tooth was considered the PAI score of the tooth. This PAI score was considered for further statistical evaluation.

### 2.9. Evaluating Radiographs

All the radiographs were done using a paralleling device with custom-made silicon keys. The two evaluators independently scored the radiographs. After the independent scoring sessions, the examiners reached an agreement on the PAI scores and whether the scores of their independent evaluations differed. The consensus scores for all the radiograph images were considered the correct score and were used for statistical analysis.

### 2.10. Statistical Analysis

All the tests were two-tailed with SPSS 25.0 (SPSS Inc., Chicago, IL, USA) at a 5% significance level. During analysis, the event of interest was the success of healing the tooth. A total of 34 variables were investigated. After identifying potential correlations between variables and the success of healing, logistic regression models were used to detect the significant outcome predictors. The odds ratio (OR) and confidence intervals (CI) were calculated.

## 3. Results

The total number of teeth treated was 90; two patients (two teeth) did not come for the follow-up. Of the 88 teeth, 84 (95.4%) were judged to have succeeded in healing, and four (4.6%) failed.

At baseline, teeth that received a PAI score > 3 were 25 (25.6%). After 48 months of follow-up, all surviving teeth were assigned a PAI score < 3 and, therefore, were considered clinically healed, according to the chosen classification (Table 2).

Overall, 34 variables were recorded for the research (Table 3). After conducting bivariate analysis, and an evaluation of current literature, the following variables were identified as potential outcome predictors: gender, age, oral hygiene, tobacco consumption, preoperative symptoms, tooth mobility, sinus tract and sealer extrusion, and included in the logistic regression.

As a result of the logistic regression, preoperative symptoms were shown to be statistically correlated with failure of endodontic treatment (*p* = 0.028) with a 6297 odds ratio.

**Figure 1:**
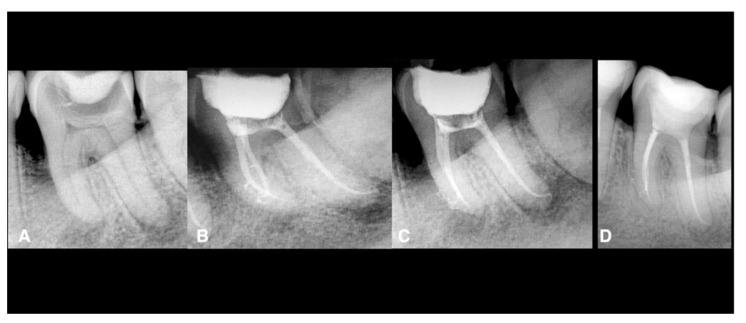
Endodontic treatment of a second mandibular left molar. The tooth was necrotically associated with periapical lesions. The treatment was done using conservative shaping and the 3D cleaning procedure, as mentioned in the Materials and Methods. (**A**) Preoperative X-ray, (**B**) postoperative X-ray, (**C**) postoperative X-ray with different angulation, (**D**) 2-year follow-up showing healing.

**Figure 2:**
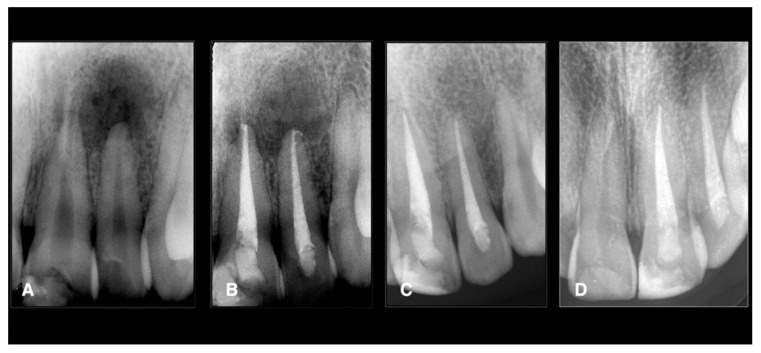
Endodontic treatment of a second maxillary left incisor. The tooth was necrotically associated with periapical lesions. The treatments were done using conservative shaping and the 3D cleaning procedure, as mentioned in the Materials and Methods. (**A**) Preoperative X-ray, (**B**) postoperative X-ray, (**C**) 1-year follow-up, (**D**) 2-year follow-up showing healing.

The four failed teeth were two maxillary premolars and two mandibular molars (these teeth did not have sealer extrusion). One maxillary premolar failed because of a vertical root fracture (Figure 1 and Figure 2) (Appendix A).

### Incidence of Pain

The incidence of pain was evaluated using a VAS scale. The preoperative evaluation indicated that 24.4% of patients reported mild pain, and 5.2% reported moderate pain. Two days after the procedure, only five patients with five molars (5.6%) reported mild pain (VAS 4). At 2, 7, and 14 days after the procedure, no patients experienced moderate or severe pain (VAS scores of 5 to 9). No pain was reported at the 1- and 2-year follow-up visits (Table 4).

## 4. Discussion

The pathological etiological factor behind pulpal and periapical infection can be traced to bacteria and their by-products [11,12]. Bearing in mind how complicated the root canal system can be, the bacteria can nest in inaccessible areas for the available technology today, rendering complete sterilization almost impossible. It is important to realize that contemporary endodontics seeks to reduce the bacterial load to a level that can promote healing via the body’s defense system [13].

Certainly, the irrigant solution typically utilized in endodontics to decrease the bacterial load and also capable of dissolving organic tissues is NaOCl [11]. Significantly, NaOCl action power can be boosted by heating to increase its temperature, leading to considerable benefits [14]. For instance, both the antibacterial activity and the ability to dissolve organic tissue will increase considerably, while the viscosity will decrease, on the other hand.

Woodmansey found that NaOCl at boiling temperatures could disintegrate the pulp tissue at a speed 210 times higher than that of the same irrigant at room temperature [15,16].

Consequently, a new approach was used in this study that implicated the heating of irrigants directly inside the root canals [1]; hence, the extraoral heating was ineffective in preserving the temperature of the pre-heated NaOCl in the root canal.

Furthermore, the new technique begins once the mechanical preparation is completed. In detail, the root canal is filled with NaOCl at a concentration of 5.25%, and then it is directly heated using the heat carrier 30/04 of System-B, followed by ultrasonic activation.

Moreover, several types of research, in vitro and ex vivo, showed promising results of this method, and ultimately, this unprecedentedly clinical study evaluated the success rate clinically.

One of the important points added to this new cleaning protocol is minimally invasive shaping [17].

Fundamentally, the accurate endodontic mechanical preparation must respect the original anatomy without modifying it. Furthermore, shaping should avoid undue dentin reduction to reduce the hazard of microcracks or stripping [18,19,20,21]. It is well established that root canal shaping procedures and rotary instrumentation can generate micro-cracks. Consequently, once the tooth is functioning and restored, it can induce complete fractures [20,22].

According to various authors, multiple nickel–titanium rotary file characteristics can directly impact microcrack formation, for instance, dimensions, heat treatments, design, cross-sectional shape, and kinematics [22,23].

The current work showed that a 95.4% success rate of healing was achieved when patients were treated with the 3D cleaning protocol combined with conservative shaping. Furthermore, out of the 88 teeth, two patients did not come for follow-up, 84 (95.4%) were healed, and four (4.6%) failed.

To explain more, in analyzing the four failed teeth, they were two maxillary premolars and two mandibular molars (these teeth did not show sealer extrusion). One maxillary premolar was diseased with vertical root fracture.

The clinical results achieved using this new protocol have been very satisfactory and promising. A similar study by Sigurdsson et al. [10], which was a 12-month prospective multicenter clinical study using conservative shaping and the GentleWave System for irrigant activation, showed a high level of success after a 12-month follow-up. This result is in accordance with our study.

Under the limitations of the current study (the lack of 3D imaging), we suggest further research with a randomized controlled clinical trial with CBCT exams to empower the results.

## 5. Conclusions

In this two-year clinical study, the cumulative success rate of healing was 95.4% when patients were treated with the 3D cleaning protocol combined with conservative shaping.

## Figures and Tables

**Table 1:** Inclusion and exclusion criteria.

Inclusion Criteria	Exclusion Criteria
Age of the patient between 18 and 70	Subject tooth having previous or attempted pulpotomy, pulpectomy, or root canal therapy
The tooth needs root canal treatment	Immunocompromised patients (i.e., corticosteroid usage)
Signed informed consent form	Any known infectious diseases (e.g., HIV, hepatitis B, hepatitis C, tuberculosis, BCE, or prion disease)
	History of cancer within the oral-maxillofacial region
	History of cancer within the past two years
	History of head and/or neck radiation therapy
	Subject tooth with mobility score ≥ 2
	Subject tooth with periodontal pocket depth ≥ 6 mm
	Subject tooth with vertical fracture or horizontal fracture extending below the cemento-enamel junction (CEJ) of the tooth
	Non-odontogenic facial pain

**Table 2:** PAI score.

	**PAI Score**						
	1 (*n*; %)	2 (*n*; %)	3 (*n*; %)	≤3 (*n*; %)	4 (*n*; %)	5 (*n*; %)	>3 (*n*; %)
Baseline *n* = 90	0	31; 34.4	36; 40.0	67; 74.4	22; 24.4	1	23; 25.6
Follow-up *n* = 88	84; 95.4	0; 0.0	0; 0.0	84 95.4	4; 4.6	0; 0.0	4; 4.6

**Table 3:** The 34 variables. Description of all assessed outcome predictors and their statistics.

	Factor	*p*-Value
1	Age	0.072
2	Gender	0.063
3	Oral hygiene	0.058
4	Diabetic history	0.853
5	Tobacco use	0.085
6	Maxillary molars	0.745
7	Right molars	0.854
8	Clinical symptoms	0.042
9	Bleeding on probing	0.207
10	Probing depth baseline	0.634
11	Sinus tract baseline	0.046
12	Fistula baseline	0.893
13	Periradicular diagnosis baseline	0.696
14	Pulp diagnosis	0.802
15	PAI score baseline	0.846
16	Number of visits	NA
17	Number of roots	0.849
18	Operator	0.568
19	Final apical diameter	0.890
20	Calcification	0.854
21	Obturation type	0.443
22	Root filling length	0.287
24	Sealer extrusion	0.391
25	Coronal seal	0.085
26	Post	0.605
27	Restoration	0.560
28	Clinical symptoms	0.822
29	Crown and bite related issues	0.763
30	Probing depth follow-up	0.225
31	Sinus tract follow-up	NA
32	Fistula follow-up	NA
33	Periradicular diagnosis follow-up	0.361
34	PAI scores follow-up	0.376

**Table 4:** Incidence of Pain.

Tooth Position	*n*	Postoperative Symptoms (VAS); *n* (%)				Follow-Up; *n* (%)	Preoperative PAI > 3; *n* (%)	Follow-Up PAI > 3; *n* (%)	Failed; *n* (%)
		1	2	3	4				
Maxillary molars	20	7 (35.0)	13 (65.0)	0 (0.0)	0 (0.0)	19 (95.0)	6 (30.0)	0 (0.0)	0 (0.0)
Maxillary premolars	30	12 (40.0)	16 (53.3)	2 (6.7)	0 (0.0)	29 (96.7)	7 (23.3)	2 (6.9)	2 (6.9)
Mandibular molars	40	7 (17.5)	10 (25.0)	18 (45.0)	5 (12.5)	40 (100.0)	10 (25.0)	2 (5.0)	2 (5.0)
**OVERALL**	90	26 (28.8)	39 (43.3)	20 (22.2)	5 (5.7)	88 (97.8)	23 (25.6)	4 (4.6)	4 (4.6)

## Data Availability

Not applicable.

## Appendix A

All data Male: 55 Female: 35 Age: all the patients were between 20 and 45 years. Oral hygiene: 65 patients had good hygiene. Tobacco use: 10 patients used it. Teeth: 20 maxillary molars, 40 mandibular molars, 30 maxillary premolars. Intraoperative data collection Twenty teeth had sealer extrusions. The working length was respected in all teeth. Postoperative data collection Post-treatment symptoms were assessed two days after the treatment using a visual analogue scale (VAS; 0 and 10) Thirteen maxillary molars: 2 Seven maxillary molars: 1 Five mandibular molars: 4 Eighteen mandibular molars: 3 Ten mandibular molars: 2 Seven mandibular molars: 1 Sixteen maxillary premolars: 2 Twelve maxillary premolars: 1 Two maxillary premolars: 3 Total number: 90 teeth Two patients (2 teeth) did not come for the follow-up. Of the 88 teeth, 84 (95%) were healed, and four (5%) failed. Four teeth failed: 2 maxillary premolars and two mandibular molars (these teeth did not have sealer extrusion). One maxillary premolar failed with vertical root fracture.

## References

[1] Amato M., Pantaleo G., Abdellatif D., Blasi A., Gagliani M.I. (2018). An in vitro evaluation of the degree of pulp tissue dissolution through different root canal irrigation protocols. J. Conserv. Dent..

[2] Teja KV Sindhu Ramesh B.G., Vasundhara K.A., Jose J., Janani K. (2022). The effect of various in-vitro and ex-vivo parameters on irrigant flow and apical pressure using manual syringe needle irrigation: Systematic review. Saudi. Dent. J..

[3] Di Spirito F., Scelza G., Fornara R., Giordano F., Rosa D., Amato A. (2022). Post-Operative Endodontic Pain Management: An Overview of Systematic Reviews on Post-Operatively Administered Oral Medications and Integrated Evidence-Based Clinical Recommendations. Healthcare.

[4] Iandolo A., Amato M., Dagna A., Poggio C., Abdellatif D., Franco V., Pantaleo G. (2018). Intracanal heating of sodium hypochlorite: Scanning electron microscope evaluation of root canal walls. J. Conserv. Dent..

[5] Teja K.V., Sindhu Ramesh S. (2020). Is a filled lateral canal—A sign of superiority?. J. Dent. Sci..

[6] Iandolo A., Abdellatif D., Amato M., Pantaleo G., Blasi A., Franco V., Neelakantan P. (2020). Dentinal tubule penetration and root canal cleanliness following ultrasonic activation of intracanal-heated sodium hypochlorite. Aust. Endod. J..

[7] Tay F.R., Gu L.S., Schoeffel G.J., Wimmer C., Susin L., Zhang K., Arun S.N., Kim J., Looney S.W., Pashley D.H. (2010). Effect of vapor lock on root canal debridement by using a side-vented needle for positive-pressure irrigant delivery. J. Endod..

[8] Munoz H.R., Camacho-Cuadra K. (2012). In Vivo efficacy of three different endodontic irrigation systems for irrigant delivery to working length of mesial canals of mandibular molars. J. Endod..

[9] Di Spirito F., Pisano M., Caggiano M., Bhasin P., Lo Giudice R., Abdellatif D. (2022). Root Canal Cleaning after Different Irrigation Techniques: An Ex Vivo Analysis. Medicina.

[10] Sigurdsson A., Garland R.W., Le K.T., Woo S.M. (2016). 12-month Healing Rates after Endodontic Therapy Using the Novel GentleWave System: A Prospective Multicenter Clinical Study. J. Endod..

[11] European Society of Endodontology (2006). Quality guidelines for endodontic treatment: Consensus report of the European Society of Endodontology. Int. Endod. J..

[12] Kakehashi S., Stanley H.R., Fitzgerald R.J. (1965). The effects of surgical exposures of dental pulps in germ-free and conventional laboratory rats. Oral Surg. Oral Med. Oral Pathol..

[13] Narayanan L.L., Vaishnavi C. (2010). Endodontic microbiology. J. Conserv. Dent..

[14] Cunningham W.T., Balekjian A.Y. (1980). Effect of temperature on collagen-dissolving ability of sodium hypochlorite endodontic irrigant. Oral Surg. Oral Med. Oral Pathol..

[15] Iandolo A., Simeone M., Orefice S., Rengo S. (2017). 3D cleaning, a perfected technique: Thermal profile assessment of heated NaOCl. G. Ital. Di Endod..

[16] Woodmansey K.F. (2005). Intracanal heating of sodium hypochlorite solution: An improbe endodontic irrigation technique. Dent. Today.

[17] Iandolo A., Abdellatif D., Pantaleo G., Sammartino P., Amato A. (2020). Conservative shaping combined with three-dimensional cleaning can be a powerful tool: Case series. J. Conserv. Dent..

[18] Tang W., Wu Y., Smales R.J. (2010). Identifying and reducing risks for potential fractures in endodontically treated teeth. J. Endod..

[19] Yuan K., Niu C., Xie Q., Jiang W., Gao L., Huang Z., Ma R. (2016). Comparative evaluation of the impact of minimally invasive preparation vs. conventional straight-line preparation on tooth biomechanics: A finite element analysis. Eur. J. Oral Sci..

[20] Liu R., Hou B.X., Wesselink P.R., Wu M.-K., Shemesh H. (2013). The incidence of root microcracks caused by 3 different single-file systems versus the ProTaper system. J. Endod..

[21] Barreto M.S., Moraes R.D.A., da Rosa R.A., Moreira C., Só M.V.R., Bier C.A.S. (2012). Vertical root fractures and dentin defects: Effects of root canal preparation, filling, and mechanical cycling. J. Endod..

[22] Devi T.P., Kaur A., Priyadarshini S., Deepak B.S., Banerjee S., Sanjeeta N. (2021). Microscopic Assessment of Dentinal Defects Induced by ProTaper Universal, ProTaper Gold, and Hyflex Electric Discharge Machining Rotary File Systems-An in vitro Study. Contemp. Clin. Dent..

[23] Iandolo A., Simeone M., Riccitiello F. (2012). The preparation of coronal isthmus is a fundamental step for long term success. G. Ital. Di Endod..

